# S100A8/S100A9 Links Diabetic Stress to Cardiac Progenitor Cell Dysfunction and Fibrotic Heart Failure: An Integrated Transcriptomic, Single‐Cell, and Functional Study

**DOI:** 10.1155/humu/1662522

**Published:** 2026-07-11

**Authors:** Yang Yang, Ge Cao

**Affiliations:** ^1^ Department of Critical Care Medicine, West China Hospital, Sichuan University/West China School of Nursing, Sichuan University, Chengdu, Sichuan, China, scu.edu.cn; ^2^ Department of Cardiovascular Surgery, West China Hospital of Sichuan University, Chengdu, Sichuan, China, wchscu.cn

**Keywords:** biomarkers, diabetes mellitus, heart failure, oncology, prognosis

## Abstract

**Background:**

Diabetes markedly increases the risk of heart failure, yet the molecular signals connecting metabolic stress, myocardial fibrosis, and impaired cardiac repair remain incompletely understood.

**Methods:**

We integrated bulk transcriptomic data from postischemic hearts (GSE26887), fibrosis‐related genes from the CTD database, protein–protein interaction (PPI) analysis, and pathway enrichment (GO, KEGG, GSEA, and GSVA) to identify candidate mediators in diabetic versus nondiabetic heart failure. Single‐cell RNA‐sequencing datasets were analyzed with Seurat to map the cellular distribution of key genes. Pan‐cancer analyses using TCGA cohorts were performed to evaluate prognostic, immune, and tumor mutation burden (TMB) correlations. Mechanistic validation was conducted in human cardiac progenitor cells (CPCs) exposed to normoglycemia or high glucose with S100A9 knockdown or overexpression, recombinant S100A8/A9, and a neutralizing S100A9 antibody. Cell viability (CCK‐8); qPCR panels for fibrosis, inflammation, and metabolic genes; ROS production (DCF‐DA and MitoSOX); and mitochondrial respiration were quantified.

**Results:**

Differential expression and PPI network analyses identified S100A8 as a fibrosis‐related hub specifically enriched in diabetic heart failure. Single‐cell mapping revealed predominant S100A8 expression in CPCs rather than mature cardiomyocytes. Pathway analyses linked S100A8 to collagen fibril organization, ECM–receptor interaction, oxidative phosphorylation, and fatty acid *β*‐oxidation. Functionally, high glucose upregulated S100A8/S100A9 and profibrotic and proinflammatory genes in CPCs, increased total and mitochondrial ROS, and reduced basal, ATP‐linked, and maximal respiration and spare capacity. S100A9 knockdown partially restored CPC proliferation, redox balance, and mitochondrial function, whereas S100A9 overexpression or recombinant S100A8/A9 further exacerbated oxidative stress and bioenergetic failure; S100A9 neutralization attenuated these effects. Pan‐cancer analyses showed that high S100A8 expression was associated with adverse prognosis, altered immune infiltration, and increased TMB in several TCGA cohorts.

**Conclusions:**

S100A8/S100A9 emerges as a central mediator linking hyperglycemia‐induced oxidative stress, metabolic inflexibility, and fibrotic reprogramming of CPCs, thereby promoting diabetic heart failure. S100A8/S100A9 may serve as a biomarker and therapeutic target at the interface of immunometabolism, cardiac regeneration, and cardio‐oncology.

## 1. Introduction

Diabetes mellitus is a chronic metabolic disorder that predisposes individuals to a wide range of microvascular and macrovascular complications throughout their lifespan [1–3]. The prevalence of Type 2 diabetes mellitus (T2DM) continues to rise at an alarming rate, with estimates predicting that the number of affected individuals will exceed 592 million by 2035, compared with 382 million in 2013 [4–6]. Both Type 1 and Type 2 diabetes share a high degree of clinical heterogeneity, and the progression of disease varies considerably among patients. Among the complications associated with diabetes, diabetic heart disease—including diabetic cardiomyopathy and heart failure—represents one of the most severe outcomes and remains a major cause of diabetes‐related morbidity and mortality [7–9].

Under physiological conditions, the heart maintains metabolic flexibility, allowing it to switch among multiple energy substrates to sustain high metabolic demand. In the healthy adult myocardium, fatty acid oxidation contributes 60%–90% of acetyl‐CoA generation, whereas glucose oxidation accounts for 10%–40% [10–12]. Due to the heart′s limited capacity for glycogen and triglyceride storage, its ability to adapt substrate utilization during increased workload is essential for maintaining contractile function. However, in diabetes, impaired insulin signaling profoundly disrupts myocardial substrate metabolism. Defective glucose uptake in skeletal muscle and excessive release of free fatty acids from adipose tissue contribute to a systemic metabolic imbalance characterized by hyperlipidemia, insulin resistance, and diminished cardiac glucose utilization. Although clinical studies consistently show reduced cardiac glucose uptake in T2DM patients, some evidence suggests that insulin may partially restore cardiac bioenergetics, highlighting the complexity of substrate regulation in diabetic hearts [13–16].

The association between diabetes and heart failure is well established. Epidemiologic data demonstrate that diabetes independently increases the risk of developing heart failure, even after accounting for traditional cardiovascular risk factors. Cardiovascular insufficiency occurs in approximately 14.5% of individuals with Type 1 diabetes and up to 35% of those with Type 2 diabetes, and heart disease continues to be the leading cause of mortality in diabetic populations. A prominent pathological feature contributing to cardiac dysfunction in diabetes is myocardial fibrosis, characterized by excessive collagen deposition and remodeling of the extracellular matrix (ECM). Fibrosis disrupts myocardial compliance and contractility, ultimately progressing to heart failure. The development of myocardial fibrosis is multifactorial, involving persistent metabolic stress, inflammation, oxidative injury, and activation of myofibroblasts. Diabetic patients exhibit a higher prevalence of both ischemic and nonischemic myocardial fibrosis, and recent studies suggest that modulation of enzymes such as Aldehyde Dehydrogenase 2 may attenuate fibrosis progression in diabetic models.

With advancements in computational biology, bioinformatics analysis has emerged as a powerful tool for identifying molecular signatures and potential biomarkers associated with cardiovascular diseases. In this study, we sought to systematically investigate the molecular intersection between diabetes and heart failure, with a specific focus on the fibrotic mechanisms underlying diabetic heart failure. We integrated transcriptomic profiling, protein–protein interaction (PPI) networks, pathway enrichment analysis, and single‐cell RNA sequencing (scRNA‐seq) to characterize cellular and molecular alterations and to identify fibrosis‐related genes that may drive diabetic cardiac injury. This comprehensive approach provides new insights into the pathogenic pathways involved in diabetic heart failure and highlights potential targets for therapeutic intervention.

## 2. Methods

### 2.1. Data Acquisition and Preprocessing

Transcriptome profiling data related to postischemic heart failure in diabetic and nondiabetic individuals were obtained from the Gene Expression Omnibus (GEO) database (https://www.ncbi.nlm.nih.gov/geo/). The microarray dataset GSE26887, generated on the GPL6244 Affymetrix Human Gene 1.0 ST Array platform, was selected for analysis. This dataset contains left ventricular tissue samples from 5 nonfailing controls, 7 diabetic patients with heart failure, and 12 nondiabetic patients with heart failure, enabling a direct comparison of transcriptional alterations associated with diabetes in the context of heart failure.

To further explore fibrosis‐related molecular mechanisms, a curated list of fibrosis‐associated genes was retrieved from the Comparative Toxicogenomics Database (CTD) (http://ctdbase.org/), which catalogs chemical–gene interactions and gene–disease associations. These fibrosis‐related genes were used for downstream integrative analysis.

### 2.2. Differential Gene Expression Analysis

Raw microarray data were subjected to preprocessing in R. Expression values were log2‐transformed and normalized using the *preprocessCore* package. Probe IDs were converted to their corresponding gene symbols based on platform annotation files. Probes mapping to multiple genes were removed, and for genes represented by multiple probes, the average expression value was used.

To minimize technical variability, batch effects were corrected using the limma package. Quality control was evaluated using boxplots to compare pre‐ and postnormalization distributions. Differential expression analysis was then performed between

1. Diabetic heart failure versus normal controls, and
2. Nondiabetic heart failure versus normal controls.

Genes with |log2 fold change| ≥ threshold and *p* < 0.05 were defined as differentially expressed. These differentially expressed genes (DEGs) were used for functional enrichment and protein interaction analyses.

### 2.3. PPI Network Construction

To elucidate potential functional interactions among DEGs, PPI networks were constructed using the STRING database (https://string‐db.org/). Only interactions with a combined score > 0.4 were considered to ensure confident biological relevance. The resulting interaction matrices were imported into Cytoscape (Version 3.7.2) for visualization and network analysis. Node degree was used to identify highly connected hub genes, which may represent key regulatory molecules in diabetic and nondiabetic heart failure.

### 2.4. Gene Set Variation Analysis (GSVA)

GSVA, a nonparametric and unsupervised approach for pathway‐level analysis, was used to assess the enrichment of predefined biological processes across samples. Gene sets were obtained from the Molecular Signatures Database (MSigDB) (v7.0). GSVA transforms gene‐level expression into enrichment scores for each pathway, enabling evaluation of functional differences between diabetic and nondiabetic heart failure groups. Enrichment scores were computed using the GSVA R package, and pathways exhibiting significant score variations were considered biologically relevant.

### 2.5. Gene Set Enrichment Analysis (GSEA)

To identify significantly enriched pathways and biological themes, GSEA was performed using gene sets from MSigDB, including Gene Ontology (GO) annotations and Kyoto Encyclopedia of Genes and Genomes (KEGG) pathways. GSEA was conducted in R following the ranking of all expressed genes based on fold change. The Top 50 significantly enriched GO terms and KEGG pathways were selected for further interpretation. The analysis provided insights into metabolic, structural, and inflammatory pathways altered in diabetic versus nondiabetic heart failure.

### 2.6. scRNA‐Seq Data Processing

scRNA‐seq data associated with cardiac tissue were downloaded from the GEO supplementary archives. Data processing was performed using the Seurat package (v3.0.2). An initial Seurat object was generated, and quality control metrics—including the number of detected genes, total UMI count, and percentage of mitochondrial transcripts—were calculated.

Cells were filtered using the following criteria:

• < 200 or > 2500 detected genes
• > 10% mitochondrial gene content

Low‐quality cells were removed, and the remaining dataset was normalized using log normalization with a scaling factor of 10,000. Highly variable genes were identified and used for principal component analysis (PCA). Statistically significant principal components were selected for downstream clustering.

Cell clustering was performed by adjusting the resolution parameter to optimize the separation of cell populations. Dimensionality reduction using UMAP allowed visualization of cellular heterogeneity. Marker genes were identified for each cluster, and clusters were annotated based on canonical cell type markers. This analysis enabled the identification of the cellular origin of key candidate genes, including S100A8.

### 2.7. Cell Culture

Human cardiac progenitor cells (CPCs) (cell line AC16) (species: *Homo sapiens*; tissue of origin: adult ventricular heart tissue) were obtained from American Type Culture Collection (ATCC) (catalog #: CRL‐3568; RRID: CVCL_4U18) on May, 2023. The cell line identity was authenticated by the supplier (ATCC) via short tandem repeat (STR) profiling. We confirm that this cell line is not known to be misidentified or contaminated. Furthermore, the cell cultures were confirmed to be free of mycoplasma contamination prior to use in experiments using a PCR‐based assay. CPCs were cultured in CPC growth medium supplemented with 10% fetal bovine serum, 1% penicillin–streptomycin, and growth factors as recommended by the manufacturer. Cells were maintained at 37°C in a humidified incubator with 5% CO_2_ and were used between Passages 3 and 8. For all experiments, CPCs were seeded in six‐well plates and allowed to reach 60%–70% confluence before treatment. To mimic normoglycemic and hyperglycemic conditions, cells were cultured in medium containing either 5.5 mM D‐glucose (normoglycemia [NG]) or 25–30 mM D‐glucose (high glucose [HG]). Osmotic controls were not used in the present study, as previously described protocols have established that the observed effects are primarily glucose‐dependent.

### 2.8. Culture and Treatment of Human CPCs

Human CPCs were obtained from a commercial source and cultured in CPC growth medium supplemented with 10% fetal bovine serum, 1% penicillin–streptomycin, and growth factors as recommended by the manufacturer. Cells were maintained at 37°C in a humidified incubator with 5% CO_2_ and were used between Passages 3 and 8. For all experiments, CPCs were seeded in six‐well plates and allowed to reach 60%–70% confluence before treatment.

To mimic normoglycemic and hyperglycemic conditions, cells were cultured in medium containing either 5.5 mM D‐glucose (NG) or 25–30 mM D‐glucose (HG). Osmotic controls were not used in the present study, as previously described protocols have established that the observed effects are primarily glucose‐dependent. CPCs were allocated into six experimental groups:

1. NG + NC: NG with nontargeting control small interfering RNA (siRNA)
2. HG + NC: HG with control siRNA
3. HG + S100A9 KD: HG with S100A9 knockdown
4. HG + S100A9 OE: HG with S100A9 overexpression
5. HG + rec S100A8/A9: HG with recombinant S100A8/A9 protein
6. HG + rec S100A8/A9 + Ab: HG with recombinant S100A8/A9 plus an S100A9‐neutralizing antibody

For *S100A9 knockdown*, CPCs were transfected with a pool of siRNAs targeting human S100A9 or with a nontargeting siRNA control using a lipid‐based transfection reagent according to the manufacturer′s instructions. For *overexpression*, cells were transfected with a plasmid encoding full‐length human S100A9 under a CMV promoter, with the corresponding empty vector as a control. Transfection efficiency was confirmed by qPCR and western blot in preliminary experiments. After 6–8 h of transfection, the medium was replaced with NG or HG medium, and cells were incubated for an additional 24 h before RNA extraction.

Recombinant human S100A8/A9 heterodimer (rec S100A8/A9) was added to the culture medium at a final concentration of 1–3 *μ*g/mL for 24 h. For neutralization studies, an anti‐S100A9 monoclonal antibody was preincubated with recombinant S100A8/A9 for 30 min at room temperature before addition to the cells; an isotype‐matched IgG served as an antibody control. Unless otherwise specified, all treatments were performed in triplicate in at least three independent biological experiments.

### 2.9. RNA Isolation and Quantitative Real‐Time PCR (qPCR)

Total RNA was extracted from CPCs using TRIzol reagent according to the manufacturer′s protocol. RNA concentration and purity were assessed spectrophotometrically, and samples with an A_260_/A_280_ ratio between 1.8 and 2.1 were used for downstream analysis. One microgram of total RNA was reverse‐transcribed into complementary DNA (cDNA) using a high‐capacity cDNA reverse transcription kit in a 20 *μ*L reaction volume.

qPCR was performed using a SYBR Green chemistry on a real‐time PCR system (e.g., Applied Biosystems StepOnePlus). Gene‐specific primers were designed to span exon–exon junctions when possible and were validated for amplification efficiency and specificity. The qPCR panel included genes related to

• S100A8/S100A9 axis: *S100A8* and *S100A9*;
• Cardiac identity and CPC function: *GATA4*, *NKX2-5*, *TNNT2* (cTnT), and *ACTN2* (*α*‐actinin);
• Fibrosis and ECM remodeling: *COL1A1*, *COL3A1*, *FN1*, *ACTA2* (*α*‐SMA), *CTGF*, *MMP2*, *MMP9*, *TIMP1*, and *TIMP2*;
• Inflammation and immune signaling: *IL6*, *TNFA*, *CCL2*, and *IL1B*;
• Metabolism and oxidative stress: *PPARGC1A* (PGC1*α*), *CPT1B*, *SOD2*, and *NOX2*.

For normalization, the housekeeping genes *GAPDH*, *ACTB*, *RPL13A*, and *18S rRNA* were quantified in parallel, and their expression stability across treatment groups was confirmed. Each qPCR reaction (10–20 *μ*L) contained diluted cDNA, SYBR Green master mix, and 0.2–0.4 *μ*M of each primer. The thermal cycling protocol consisted of an initial denaturation at 95°C, followed by 40 cycles of denaturation, annealing, and extension at gene‐specific temperatures. Melting‐curve analysis was performed to verify the specificity of amplification.

**Figure 1:**
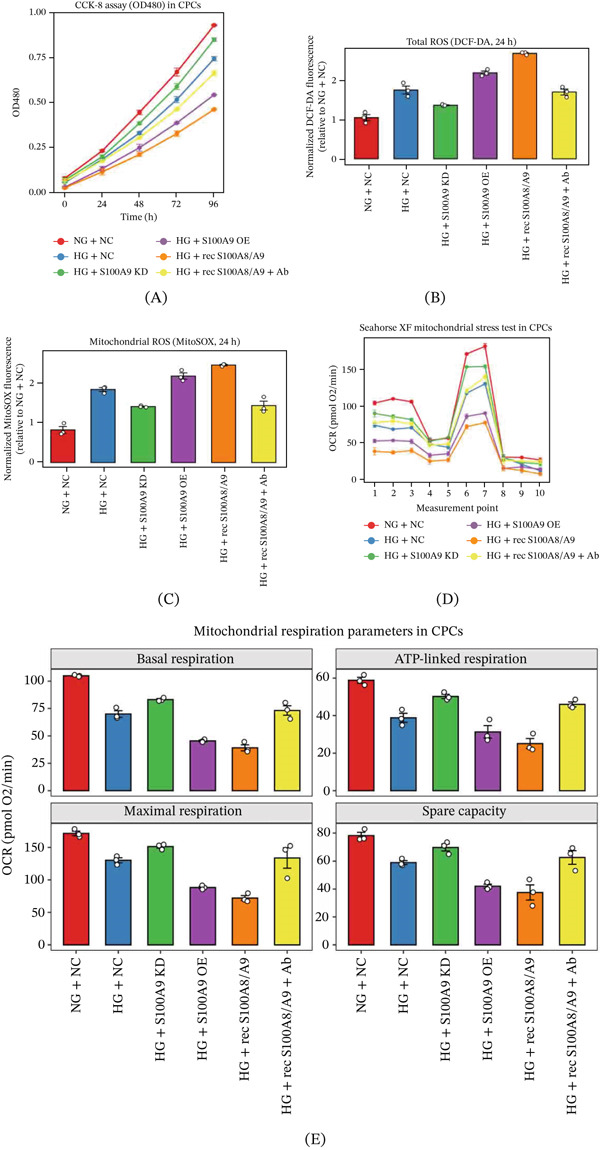
S100A8/S100A9 promotes oxidative stress and impairs mitochondrial bioenergetics in CPCs under high‐glucose conditions. (A) CCK‐8 assay showing time‐dependent changes in CPC viability under normoglycemic (NG + NC) and high‐glucose conditions with the indicated S100A9 perturbations and recombinant S100A8/A9 treatments. Absorbance at 480 nm (OD480) was measured at 0, 24, 48, 72, and 96 h. High glucose reduced cell growth compared with NG, which was partially rescued by S100A9 knockdown and further suppressed by S100A9 overexpression or recombinant S100A8/A9. Neutralization of S100A9 attenuated the inhibitory effect of recombinant S100A8/A9. (B) Total intracellular ROS levels in CPCs after 24 h of treatment, assessed by DCF‐DA fluorescence and normalized to the NG + NC group. High glucose increased ROS production, which was reduced by S100A9 knockdown and enhanced by S100A9 overexpression or recombinant S100A8/A9. S100A9 neutralization partially reversed the ROS increase induced by recombinant S100A8/A9. (C) Mitochondrial ROS levels measured by MitoSOX Red fluorescence after 24 h. The pattern paralleled total ROS, with the highest mitochondrial ROS observed in the HG + S100A9 OE and HG + rec S100A8/A9 groups and partial normalization in the HG + S100A8/A9 + Ab group. (D) Seahorse XF mitochondrial stress test in CPCs. OCR traces show basal respiration, oligomycin‐induced suppression, FCCP‐induced maximal respiration, and rotenone/antimycin A–inhibited nonmitochondrial respiration for each treatment group. High glucose depressed OCR throughout the assay; S100A9 knockdown shifted the curve toward the normoglycemic profile, whereas S100A9 overexpression and recombinant S100A8/A9 further reduced OCR. S100A9 neutralization mitigated the effects of recombinant S100A8/A9. (E) Quantification of mitochondrial respiration parameters derived from the Seahorse assay: basal respiration, ATP‐linked respiration, maximal respiration, and spare respiratory capacity. All four parameters were reduced by high glucose, partially restored by S100A9 knockdown, and further decreased by S100A9 overexpression or recombinant S100A8/A9, with partial recovery in the recombinant S100A8/A9 + Ab group.

All samples were run in technical triplicate. Relative mRNA expression levels were calculated using the 2^(‐*ΔΔ*Ct) method, with the NG + NC group serving as the calibrator. For the focused panel shown in Figure 1A, results are presented as mean ± SEM of three independent experiments. For the extended panel (Figure 1B), normalized expression values are displayed as bar graphs with error bars representing the SEM across biological replicates.

### 2.10. Cell Viability Assay (CCK‐8)

Cell viability and proliferation of CPCs under different treatments were evaluated using a CCK‐8 assay. CPCs were seeded in 96‐well plates at a density of 3–5 × 10^3^ cells/well and allowed to attach overnight in complete growth medium. Cells were then exposed to either NG (5.5 mM D‐glucose) or HG (25–30 mM D‐glucose) conditions and assigned to the following groups:

1. NG + NC (NG + nontargeting control siRNA),
2. HG + NC (HG + control siRNA),
3. HG + S100A9 KD (HG + S100A9 siRNA),
4. HG + S100A9 OE (HG + S100A9 overexpression plasmid),
5. HG + rec S100A8/A9 (HG + recombinant S100A8/A9 heterodimer),
6. HG + rec S100A8/A9 + Ab (HG + recombinant S100A8/A9 plus S100A9‐neutralizing antibody).

Transfection and recombinant protein/antibody treatments were performed as described in the qPCR experiments. At 0, 24, 48, 72, and 96 h after treatment initiation, 10 *μ*L of CCK‐8 reagent was added to each well containing 100 *μ*L culture medium and incubated for 1–2 h at 37°C. Absorbance was measured at 480 nm (OD480) using a microplate reader. Blank wells containing medium and CCK‐8 without cells were used for background correction. OD480 values were normalized to the 0 h time point or to the NG + NC group as indicated. Each condition was tested in at least three independent biological replicates, with three to five technical replicates per experiment.

### 2.11. Measurement of Intracellular and Mitochondrial Reactive Oxygen Species (ROS)

Total intracellular ROS levels were assessed using 2 ^′^,7 ^′^‐dichlorodihydrofluorescein diacetate (DCFH‐DA), and mitochondrial ROS were measured using MitoSOX Red. CPCs were seeded in black 96‐well plates with clear bottoms and treated under the same NG/HG and S100A8/S100A9 conditions as described above for 24 h.

For total ROS, cells were washed twice with warm PBS and incubated with 10 *μ*M DCFH‐DA diluted in serum‐free medium for 20–30 min at 37°C in the dark. After incubation, cells were rinsed with PBS to remove excess probe and maintained in phenol red–free medium. Fluorescence was measured with a microplate reader at Ex 485 nm/Em 525 nm.

For mitochondrial ROS, cells were incubated with 5 *μ*M MitoSOX Red in HBSS for 10–15 min at 37°C and washed with PBS, and fluorescence was recorded at Ex 510 nm/Em 580 nm. In some experiments, representative images were obtained using a fluorescence microscope with identical exposure settings across groups.

For both assays, background fluorescence from cell‐free wells was subtracted, and fluorescence intensities were normalized to the NG + NC group, which was set to 1.0. Each condition was measured in at least three independent biological experiments.

### 2.12. Seahorse XF Mitochondrial Stress Test

Mitochondrial respiration was analyzed using a Seahorse XF extracellular flux analyzer (XF24 or XF96, Agilent). CPCs were seeded in Seahorse XF cell culture microplates at a density of 3–5 × 10^4^ cells/well and allowed to adhere overnight. Cells were then exposed to NG or HG medium and the six treatment conditions (NG + NC, HG + NC, HG + S100A9 KD, HG + S100A9 OE, HG + rec S100A8/A9, and HG + rec S100A8/A9 + Ab) for ~24 h.

On the day of the assay, cells were washed and incubated in Seahorse XF assay medium (unbuffered DMEM supplemented with 10 mM glucose, 1 mM sodium pyruvate, and 2 mM glutamine, pH 7.4) for 45–60 min at 37°C in a non‐CO_2_ incubator. The XF Mito Stress Test was performed by sequential injection of

• Oligomycin (1 *μ*M) to inhibit ATP synthase,
• FCCP (0.5–1 *μ*M) to uncouple oxidative phosphorylation and reveal maximal respiration,
• Rotenone/Antimycin A (0.5 *μ*M each) to inhibit Complexes I and III and determine nonmitochondrial respiration.

Oxygen consumption rate (OCR) was recorded at multiple time points before and after each injection. Basal respiration, ATP‐linked respiration, maximal respiration, and spare respiratory capacity were calculated using standard Seahorse protocols:

• Basal respiration = baseline OCR − nonmitochondrial OCR
• ATP − linked respiration = baseline OCR − OCR after oligomycin
• Maximal respiration = OCR after FCCP − nonmitochondrial OCR
• Spare capacity = maximal respiration − basal respiration

Each treatment group contained at least three to five wells per experiment, and data represent ≥ 3 independent biological repeats. OCR values are expressed as picomoles of oxygen per minute (pmol O_2_/min) and, when indicated, normalized to cell number or protein content.

### 2.13. Statistical Analysis

All statistical analyses were conducted using R (Version 3.6) software. Statistical tests were two‐sided, and a *p* value < 0.05 was considered statistically significant.

## 3. Results

### 3.1. Identification of Key Genes Associated With Heart Failure in Diabetic and Nondiabetic Conditions

**Figure 2:**
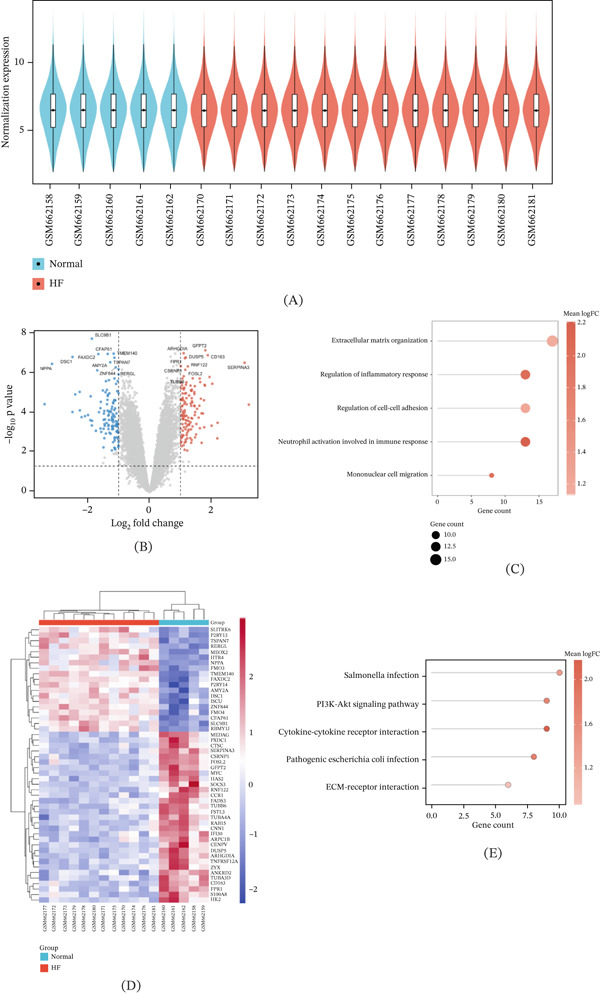
Differential expression and enrichment analysis between normal and nondiabetic heart failure samples. (A) Boxplots showing normalized expression distributions across samples from normal and nondiabetic heart failure cohorts after preprocessing and batch correction. (B, C) Volcano plot and heat map illustrating differentially expressed genes (DEGs) between normal and nondiabetic heart failure tissues. Red and blue represent upregulated and downregulated genes, respectively. (D) Gene Ontology (GO) enrichment analysis of the identified DEGs, highlighting biological processes significantly dysregulated in nondiabetic heart failure. (E) Kyoto Encyclopedia of Genes and Genomes (KEGG) pathway enrichment analysis revealing key signaling pathways involved in nondiabetic heart failure.

To explore the molecular mechanisms underlying the interplay between diabetes and heart failure, we first analyzed the transcriptomic dataset GSE26887, which contains mRNA expression profiles from 5 normal left ventricular tissues, 7 diabetic patients with postischemic heart failure, and 12 nondiabetic patients with postischemic heart failure. After data preprocessing and normalization, differential expression analysis revealed substantial transcriptional alterations in diabetic heart failure compared with normal controls. In total, 206 DEGs were identified, including 94 upregulated and 112 downregulated genes (Figures 2A, 2B, and 2C). GO functional enrichment indicated that these DEGs were significantly involved in inflammatory response regulation, cell–cell adhesion, mononuclear cell chemotaxis, neutrophil activation, and ECM remodeling, highlighting an inflammatory–fibrotic signature characteristic of diabetic myocardial dysfunction. KEGG analysis further implicated pathways such as PI3K–Akt signaling, cytokine–cytokine receptor interaction, ECM–receptor interaction, and infection‐related signaling (e.g., *Salmonella* and pathogenic *E. coli* pathways), suggesting activation of immune–metabolic stress networks (Figure 2D,E).

**Figure 3:**
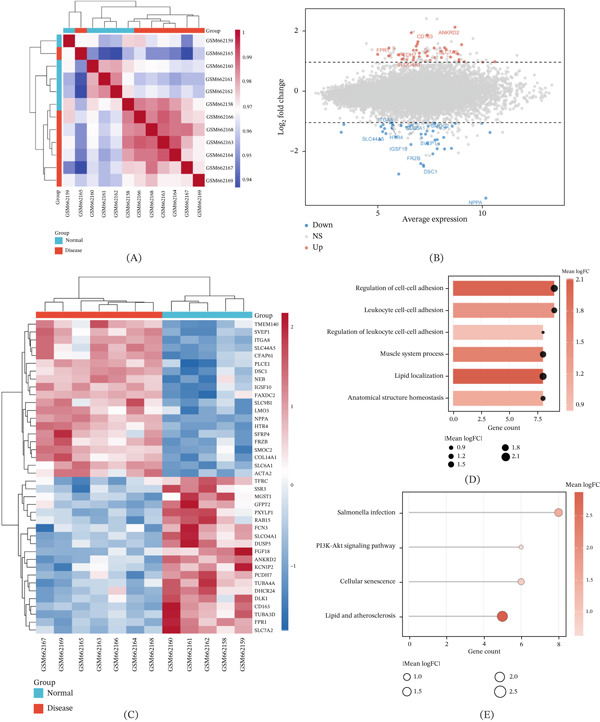
Differential expression and enrichment analysis between normal and diabetic heart failure samples. (A) Boxplots displaying normalized gene expression distributions between normal hearts and diabetic heart failure tissues. (B, C) Volcano plot and heat map of DEGs identified between normal and diabetic heart failure cohorts, demonstrating significant transcriptomic alterations driven by diabetes. (D) GO enrichment analysis of DEGs associated with diabetic heart failure, indicating biological functions most affected by hyperglycemic stress. (E) KEGG pathway enrichment analysis showing major pathways implicated in diabetic heart failure progression.

In contrast, the nondiabetic heart failure group showed a partially distinct molecular pattern. We identified 265 DEGs, including 136 upregulated and 129 downregulated genes (Figures 3A, 3B, and 3C). GO enrichment highlighted associations with lipid localization, muscle system processes, regulation of leukocyte adhesion, and maintenance of anatomical structure, whereas KEGG analysis again identified PI3K–Akt signaling, infection‐related pathways, and lipid/atherosclerosis‐associated pathways (Figure 3D,E). These results indicate both shared and diabetes‐specific transcriptional programs in the progression of heart failure, suggesting that metabolic disturbances amplify inflammatory and ECM‐related processes.

### 3.2. PPI Network Identifies Fibrosis‐Related S100A8 as a Key Node in Diabetic Heart Failure

**Figure 4:**
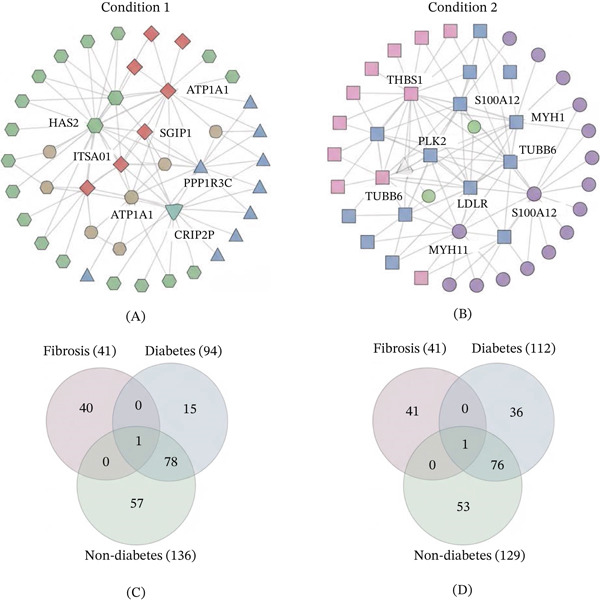
Protein–protein interaction network analysis and association with fibrosis‐related genes. (A) Protein–protein interaction (PPI) network constructed from DEGs in the nondiabetic heart failure cohort, with node size representing interaction counts. (B) PPI network of DEGs in the diabetic heart failure cohort, highlighting highly connected hub genes. (C, D) Venn diagrams illustrating the overlap between DEGs and fibrosis‐associated genes retrieved from the CTD database, demonstrating key fibrosis‐related genes enriched in both nondiabetic and diabetic heart failure.

We next constructed PPI networks to evaluate the connectivity and functional relevance of DEGs in both diabetic and nondiabetic heart failure. In the nondiabetic group, 39 genes showed high interaction degrees, including central nodes such as MYC, CD44, MYH6, SPP1, TIMP1, ACTA2, CCL2, and THBS1, each exhibiting > 25 connections (Figure 4A). These hub genes highlighted coordinated changes involving ECM composition, fibroblast activation, inflammation, and cardiac contractile function.

In diabetic heart failure, the interaction landscape showed even stronger clustering of inflammatory and fibrotic mediators. Key high‐degree nodes included IL6 (66 interactions), MYC (46), CCL2 (44), SPP1 (34), ACTA2 (32), and KIT, SERPINE1, and TFRC (all ≥ 26 interactions) (Figure 4B). Importantly, when DEGs were overlapped with fibrosis‐related genes from the CTD, the calcium‐binding protein S100A8 emerged as a consistent fibrosis‐associated candidate specifically enriched in *diabetic* heart failure (Figure 4C,D). This finding suggests that S100A8 may act as a diabetes‐specific molecular mediator that links inflammation, ECM remodeling, and myocardial dysfunction.

### 3.3. Single‐Cell Transcriptomics Identifies CPC‐Specific Expression of S100A8

**Figure 5:**
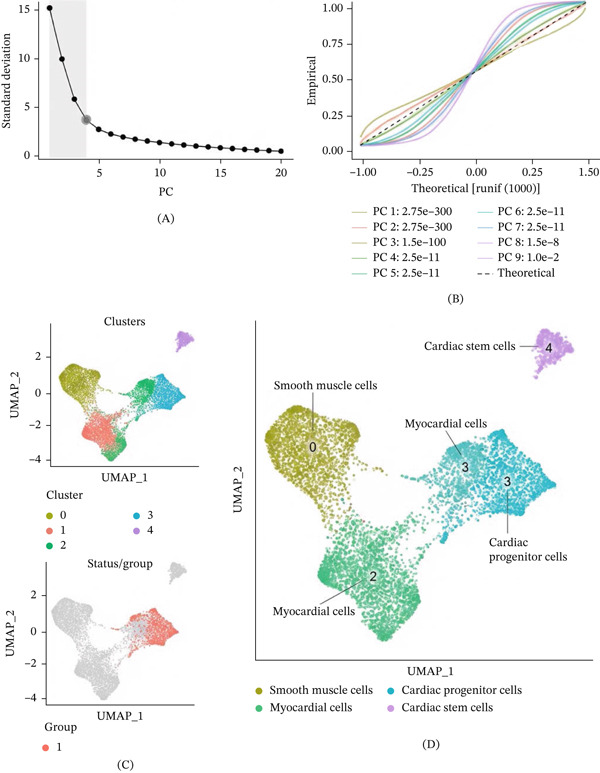
Dimensionality reduction, clustering, and annotation of single‐cell populations. (A) ElbowPlot used to select principal components contributing most significantly to the dataset variance. (B) JackStrawPlot showing statistical significance of each principal component and guiding PC selection. (C) UMAP visualization of cell clusters, revealing major cellular subpopulations in heart tissue. (D) Manually annotated cell‐type map based on canonical marker genes for each cluster.

We performed PCA and selected significant components for clustering. UMAP projection clearly separated major myocardial cell types (Figures 5A, 5B, and 5C). Cell identities were annotated using canonical markers, resulting in clusters representing cardiomyocytes, fibroblasts, endothelial cells, immune cells, and CPCs (Figure 5D).

**Figure 6:**
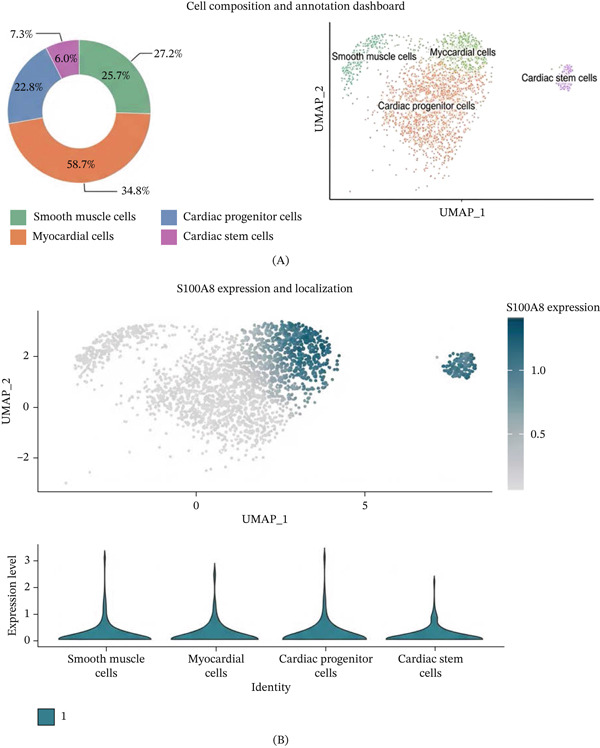
Cell composition and S100A8 expression across cardiac cell types. (A) Proportional distribution of annotated cell populations within the analyzed heart tissue samples. (B) S100A8 expression levels mapped across different cardiac cell types, highlighting its preferential enrichment in specific populations.

Surprisingly, S100A8 expression was highly enriched in CPCs, rather than in mature cardiomyocytes or fibroblasts (Figure 6A,B). This suggests a previously underappreciated role for S100A8 in cardiac regeneration and progenitor cell dysfunction, potentially contributing to impaired myocardial repair in diabetes‐associated heart failure.

### 3.4. Functional Pathways Associated With S100A8 in Diabetic and Nondiabetic Heart Failure

**Figure 7:**
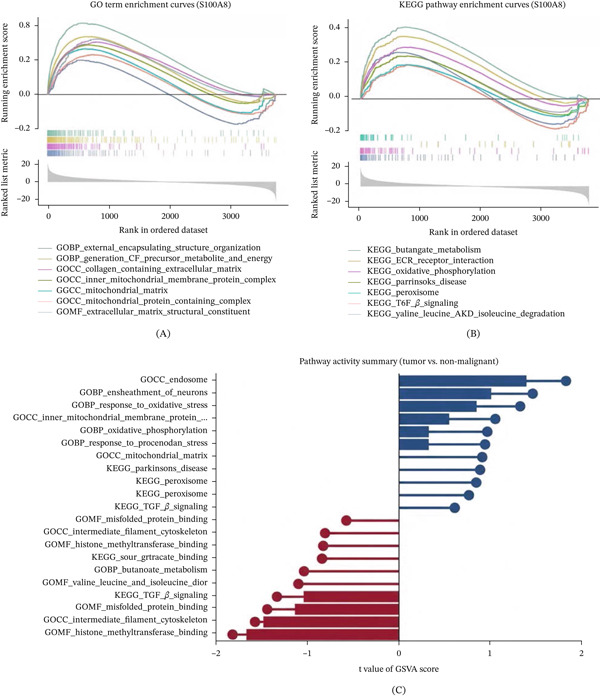
Pathway enrichment analysis of diabetic and non‐diabetic heart failure. (A) GSEA enrichment results based on GO biological processes, identifying pathways significantly enriched between conditions. (B) GSEA enrichment results based on the KEGG database, demonstrating metabolic and structural pathways associated with disease progression. (C) GSVA heat map showing pathway activity differences across samples, indicating functional divergence between diabetic and nondiabetic heart failure.

To elucidate pathway‐level mechanisms linking S100A8 to cardiac pathology, GSEA and GSVA analyses were performed. GO‐based GSEA revealed enrichment of pathways involved in collagen fibril organization, ECM structural integrity, oxidative phosphorylation, and fatty acid *β*‐oxidation (Figure 7A), indicating that mitochondrial dysfunction and fibrotic remodeling are common features of both diabetic and nondiabetic heart failure.

KEGG‐based GSEA highlighted metabolic and signaling pathways, including oxidative phosphorylation, ECM–receptor interaction, valine/leucine/isoleucine degradation, butanoate metabolism, TGF‐*β* signaling, and focal adhesion (Figure 7B). These pathways reinforce the notion that S100A8 may influence both metabolic rewiring and fibrosis‐associated signaling cascades.

GSVA revealed clear molecular differences between diabetic and nondiabetic heart failure (Figure 7C). Diabetic heart failure showed enrichment in pathways related to oxidative stress, endosomal transport, cell cycle activation, immune response, and metal ion binding, reflecting heightened inflammatory and metabolic burden. In contrast, nondiabetic heart failure showed activation of pathways associated with vesicle membrane dynamics, histone methyltransferase binding, glycosyltransferase activity, ER function, and intermediate filament cytoskeleton, suggesting distinct structural and metabolic remodeling independent of hyperglycemia.

### 3.5. Pan‐Cancer Analysis Identifies S100A8 as a Prognostic and Immunomodulatory Biomarker

**Figure 8:**
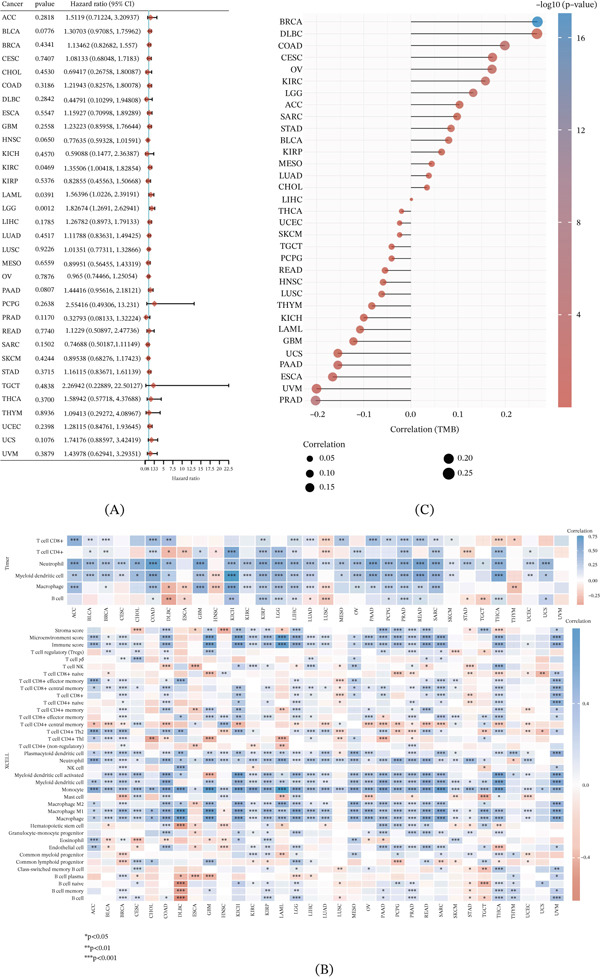
Pan‐cancer analysis of S100A8 across TCGA cohorts. (A) Prognostic analysis showing associations between S100A8 expression and patient survival outcomes across different cancer types. (B) Immune infiltration analysis depicting correlations between S100A8 expression and infiltration levels of various immune cell subsets. (C) Tumor mutation burden (TMB) analysis showing correlations between S100A8 expression and TMB across multiple cancers, suggesting its role in genomic instability and immune modulation.

Given the established involvement of S100A8/A9 in inflammation and tumor biology, we extended our analysis to a pan‐cancer framework. Survival analysis revealed that high S100A8 expression correlated with poor prognosis in multiple cancers, including kidney renal clear cell carcinoma (TCGA‐KIRC), acute myeloid leukemia (TCGA‐LAML), and lower grade glioma (TCGA‐LGG) (Figure 8A).

Immune infiltration analysis showed significant correlations between S100A8 expression and multiple immune cell populations—especially M1/M2 macrophages, stromal components, and tumor microenvironment scores (Figure 8B). Furthermore, S100A8 expression positively correlated with tumor mutation burden (TMB) in cancers such as TCGA‐BRCA, TCGA‐DLBC, TCGA‐COAD, TCGA‐CESC, and TCGA‐OV (Figure 8C). These findings suggest that S100A8 acts as a broad regulator of immune–inflammatory activity, genomic instability, and tumor aggressiveness, consistent with its role in chronic inflammation.

### 3.6. S100A9 Modulates Fibrotic, Inflammatory, and Cardiac Gene Expression in CPCs Exposed to HG

**Figure 9:**
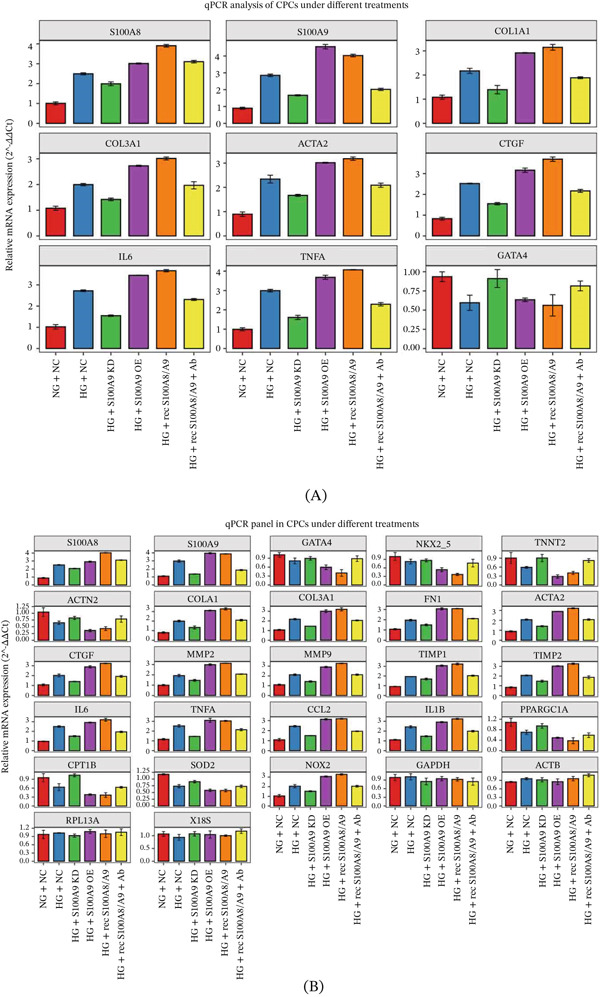
S100A9 regulates fibrotic, inflammatory, and cardiac gene expression in CPCs under high‐glucose conditions. (A) Focused qPCR analysis of S100A8/S100A9, fibrosis‐related genes (*COL1A1*, *COL3A1*, *ACTA2*, and *CTGF*), inflammatory cytokines (*IL6* and *TNFA*), and the cardiac transcription factor *GATA4* in human cardiac progenitor cells (CPCs) cultured under different conditions. Cells were exposed to normoglycemia (NG, 5.5 mM glucose) or high glucose (HG, 25–30 mM glucose) and allocated into six groups: NG + NC (normoglycemia + nontargeting siRNA), HG + NC (high glucose + nontargeting siRNA), HG + S100A9 KD (high glucose + S100A9 knockdown), HG + S100A9 OE (high glucose + S100A9 overexpression), HG + rec S100A8/A9 (high glucose + recombinant S100A8/A9 heterodimer), and HG + rec S100A8/A9 + Ab (high glucose + recombinant S100A8/A9 plus S100A9‐neutralizing antibody). High glucose increased S100A8/S100A9 expression and upregulated fibrotic and inflammatory markers while suppressing *GATA4*; S100A9 knockdown partially reversed these effects, whereas S100A9 overexpression or recombinant S100A8/A9 further exacerbated them. Neutralization of S100A9 attenuated the actions of recombinant S100A8/A9. (B) Extended qPCR panel of CPCs assessing genes related to cardiac identity (*GATA4*, *NKX2-5*, *TNNT2*, and *ACTN2*), extracellular matrix remodeling (*COL1A1*, *COL3A1*, *FN1*, *ACTA2*, *CTGF*, *MMP2*, *MMP9*, *TIMP1*, and *TIMP2*), inflammation and immune signaling (*IL6*, *TNFA*, *CCL2*, and *IL1B*), and cellular metabolism/oxidative stress (*PPARGC1A*, *CPT1B*, *SOD2*, and *NOX2*), under the same six treatment conditions as in (A). High glucose induced a profibrotic, proinflammatory, and metabolically unfavorable expression profile, which was ameliorated by S100A9 knockdown and aggravated by S100A9 overexpression or recombinant S100A8/A9. Cotreatment with the S100A9‐neutralizing antibody partially normalized these changes. The housekeeping genes *GAPDH*, *ACTB*, *RPL13A*, and *18S* remained stable across groups and were used for normalization.

To validate the bioinformatic findings at the cellular level, we first examined the expression of S100A8/S100A9 and fibrosis‐related genes in human CPCs exposed to HG. Compared with normoglycemic controls (NG + NC), HG markedly increased the mRNA levels of S100A8 and S100A9, as well as the profibrotic markers COL1A1, COL3A1, ACTA2, and CTGF (Figure 9A). In parallel, the proinflammatory cytokines IL6 and TNFA were significantly upregulated in the HG group, whereas the cardiac transcription factor GATA4 was reduced, indicating impaired CPC phenotype under diabetic‐like conditions (Figure 1A). To clarify the relationship between S100A9 silencing and S100A8/S100A9‐axis expression, we performed qPCR validation in AC16 cells under normoglycemic and HG conditions. HG markedly increased the mRNA abundance of both S100A9 and S100A8 compared with the normoglycemic control. Importantly, transfection with siS100A9 under HG conditions strongly suppressed S100A9 expression, confirming efficient knockdown. In parallel, S100A8 expression was also reduced following S100A9 silencing, although the magnitude of reduction was less pronounced than that observed for S100A9 itself. These data support functional coupling within the S100A8/A9 axis and indicate that perturbation of S100A9 not only abrogates its own induction but is also accompanied by partial attenuation of S100A8 transcription under HG stress (Figure S1).

Silencing S100A9 under HG (HG + S100A9 KD) partially normalized this expression pattern. S100A9 knockdown led to a reduction of S100A8/S100A9 transcripts and significantly decreased collagen genes (COL1A1 and COL3A1), ACTA2, CTGF, IL6, and TNFA compared with HG alone while restoring GATA4 expression toward normoglycemic levels (Figure 9A). In contrast, S100A9 overexpression (HG + S100A9 OE) further amplified the HG‐induced upregulation of S100A8/S100A9, fibrotic genes, and inflammatory cytokines and exacerbated the suppression of GATA4 (Figure 9A). Treatment with recombinant S100A8/A9 protein (HG + rec S100A8/A9) produced a similar or even more pronounced profibrotic and proinflammatory profile, which was attenuated by coadministration of an S100A9‐neutralizing antibody (HG + rec S100A8/A9 + Ab) (Figure 9A). Together, these data support a causal role for S100A9 in driving inflammatory and fibrotic gene reprogramming in CPCs under hyperglycemic stress.

To obtain a broader view of S100A9‐dependent transcriptional changes, we extended the qPCR panel to include genes related to cardiac identity, ECM remodeling, inflammation, and cellular metabolism (Figure 1B). HG significantly downregulated the cardiac markers GATA4, NKX2‐5, TNNT2, and ACTN2 and metabolic regulators PPARGC1A, CPT1B, and SOD2 while upregulating FN1, MMP2, MMP9, TIMP1, TIMP2, CCL2, IL1B, and NOX2 (Figure 1B). S100A9 knockdown partially rescued cardiac and metabolic gene expression and reduced ECM‐ and inflammation‐related transcripts, whereas S100A9 overexpression or recombinant S100A8/A9 treatment further aggravated these alterations (Figure 9B). Notably, S100A9 neutralization in the presence of recombinant S100A8/A9 reversed a substantial proportion of these changes, indicating that the S100A8/A9–S100A9 axis is a key upstream regulator of CPC fibrotic and inflammatory programming. Expression of the housekeeping genes GAPDH, ACTB, RPL13A, and 18S remained stable across all conditions, supporting the robustness of the normalization strategy (Figure 9B).

### 3.7. S100A8/S100A9 Promotes Oxidative Stress and Impairs Mitochondrial Bioenergetics in CPCs Under HG Conditions

To determine whether the S100A8/S100A9 axis affects CPC viability under diabetic conditions, we first assessed cell proliferation by CCK‐8 assay. In normoglycemic CPCs (NG + NC), OD480 values increased steadily from 0 to 96 h, reflecting robust cell growth (Figure 1A). HG significantly suppressed CPC proliferation across all time points compared with NG + NC. S100A9 knockdown partially rescued this defect, whereas S100A9 overexpression further reduced OD480, indicating more pronounced growth inhibition. Treatment with recombinant S100A8/A9 protein resulted in the greatest decrease in proliferation, while cotreatment with an S100A9‐neutralizing antibody partially restored growth, supporting a detrimental effect of S100A8/S100A9 signaling on CPC survival and expansion under hyperglycemic stress (Figure 1A).

We next examined whether S100A8/S100A9 modulates redox homeostasis in CPCs. Total intracellular ROS, measured by DCF‐DA fluorescence, were significantly elevated in the HG + NC group compared with NG + NC (Figure 1B). S100A9 knockdown reduced DCF fluorescence toward control levels, whereas S100A9 overexpression and recombinant S100A8/A9 treatment further increased ROS accumulation. Notably, neutralization of S100A9 markedly attenuated the ROS burst induced by recombinant S100A8/A9 (Figure 1B). A similar pattern was observed for mitochondrial ROS measured by MitoSOX: HG induced a marked increase in mitochondrial superoxide, which was exacerbated by S100A9 overexpression or recombinant S100A8/A9 and mitigated by S100A9 knockdown or antibody treatment (Figure 1C). These data indicate that S100A8/S100A9 strongly amplifies both cytosolic and mitochondrial oxidative stress in CPCs exposed to HG.

To evaluate the impact of S100A8/S100A9 on mitochondrial bioenergetics, we performed a Seahorse XF mitochondrial stress test. In NG + NC cells, OCR curves displayed a typical profile, with stable basal respiration, an oligomycin‐induced decrease, a pronounced increase after FCCP reflecting maximal respiration, and a marked drop following rotenone/Antimycin A administration (Figure 1D). HG globally suppressed OCR throughout the assay. S100A9 knockdown shifted the OCR trace upward toward the normoglycemic profile, whereas S100A9 overexpression and recombinant S100A8/A9 further depressed OCR at baseline, after FCCP, and following subsequent injections. Neutralizing S100A9 in the presence of recombinant S100A8/A9 partially restored OCR, indicating that S100A8/A9‐mediated mitochondrial dysfunction is largely S100A9‐dependent (Figure 1D).

Quantitative analysis of mitochondrial respiration parameters confirmed these observations (Figure 1E). Compared with NG + NC, HG significantly reduced basal respiration, ATP‐linked respiration, maximal respiration, and spare respiratory capacity. S100A9 knockdown partially rescued all four parameters, whereas S100A9 overexpression and recombinant S100A8/A9 induced profound decreases, particularly in maximal respiration and spare respiratory capacity. Cotreatment with S100A9‐neutralizing antibody improved mitochondrial function relative to recombinant S100A8/A9 alone, although values remained below those of NG + NC (Figure 1E). Collectively, these findings demonstrate that S100A8/S100A9 aggravates HG‐induced oxidative stress and severely compromises mitochondrial respiratory capacity in CPCs, providing a mechanistic link between S100A8 signaling, impaired cellular metabolism, and reduced reparative potential in diabetic heart failure.

## 4. Discussion

Beyond identifying S100A8 as a potential fibrosis‐associated gene in diabetic heart failure, our findings also highlight broader biological processes that may bridge metabolic dysregulation and structural cardiac remodeling. Diabetes induces chronic systemic inflammation, altering innate immune responses, macrophage polarization, and neutrophil activation—all of which are tightly linked to myocardial injury and fibrotic remodeling. S100A8, often forming the S100A8/A9 heterodimer (calprotectin), is known to function as a damage‐associated molecular pattern (DAMP) that amplifies inflammatory cascades through TLR4 and RAGE signaling pathways. These mechanisms are highly relevant to diabetic cardiomyopathy, where low‐grade inflammation persists for years before clinical heart failure becomes apparent.

In this context, our integrated bioinformatics analysis suggests that S100A8 may serve not only as a biomarker but also as a functional mediator of diabetic cardiac remodeling. The enrichment of pathways related to ECM organization, oxidative phosphorylation, fatty acid metabolism, and immune activation supports the possibility that S100A8 contributes to both metabolic inflexibility and structural deterioration of diabetic myocardium. Chronic elevation of S100A8 may exacerbate oxidative stress or mitochondrial dysfunction, further impairing CPC survival and inhibiting endogenous repair mechanisms.

Furthermore, the scRNA‐seq results provide an additional layer of mechanistic insight. The predominant expression of S100A8 in CPCs suggests a potentially underappreciated link between stem/progenitor cell dysfunction and metabolic disease–related cardiac injury. CPCs are essential for maintaining myocardial homeostasis and contributing to tissue regeneration after injury. Persistent exposure to hyperglycemia and inflammation, possibly amplified by S100A8 signaling, may compromise the regenerative niche, thereby accelerating fibrosis and ventricular dysfunction. This relationship aligns with emerging evidence that metabolic disease induces progenitor cell senescence, epigenetic remodeling, and impaired paracrine signaling.

Another important observation from our enrichment analyses is the consistent involvement of ECM–receptor interaction and TGF‐*β* signaling pathways in diabetic heart failure. The TGF‐*β* pathway is a central driver of fibroblast activation, ECM deposition, and myofibroblast differentiation. The convergence of S100A8‐associated pathways with TGF‐*β*‐mediated processes highlights a potential crosstalk between inflammatory mediators and canonical fibrosis pathways. This suggests that S100A8 may prime the myocardium for exaggerated fibrotic responses by modulating immune–fibroblast interactions or altering ECM homeostasis.

Notably, our pan‐cancer analysis revealed that S100A8 is significantly associated with immune infiltration, macrophage polarization, and TMB in several cancer types. These findings underscore its broader role as an immunomodulatory molecule, reinforcing the idea that S100A8‐driven inflammation may be a common thread linking multiple chronic diseases, including diabetes and heart failure. Given the rising interest in metabolic–immune interactions, future studies should explore whether S100A8 could serve as a therapeutic target capable of modulating both metabolic dysfunction and inflammatory cardiac remodeling.

Despite these promising insights, our study has several limitations. First, the transcriptomic data analyzed were derived from relatively small patient cohorts, which may introduce sampling bias. Second, while bioinformatics analysis provides robust correlations, it cannot establish causality. Functional experiments—such as modulation of S100A8/S100A9 expression in diabetic models—are needed to confirm the mechanistic pathways proposed here. Additionally, the role of CPCs in adult human myocardial repair remains debated; thus, the biological relevance of S100A8 expression in these cells requires validation through in vitro and in vivo studies.

Nevertheless, the integration of bulk transcriptomics, single‐cell sequencing, and pathway enrichment provides a comprehensive framework for understanding the molecular landscape of diabetic heart failure. Our findings illuminate the potential role of S100A8 as a mediator of fibrosis and metabolic dysregulation, offering new avenues for therapeutic intervention. In light of the growing global burden of diabetes and heart failure, further investigation into S100A8‐targeted therapies—such as neutralizing antibodies, small‐molecule inhibitors, or modulation of upstream regulators—may hold significant clinical value. Although our single‐cell reanalysis suggests that S100A8‐enriched signals are present within a cardiac‐resident cell population showing partial progenitor‐like/interstitial features, we acknowledge that the precise lineage identity of this cluster cannot yet be established with complete certainty. In particular, because S100A8/S100A9 are also well‐recognized markers of infiltrating myeloid populations, including monocytes and neutrophils, the possibility of overlap with immune‐lineage signatures must be considered carefully. Therefore, the current data should not be interpreted as definitive proof of a bona fide CPC‐specific source of S100A8. Instead, our results support the presence of an S100A8‐associated cellular compartment within the diseased cardiac microenvironment, while the exact lineage composition and cellular origin require further validation using more stringent marker‐based annotation, orthogonal lineage‐resolved approaches, and ideally tissue‐level spatial or immunophenotypic confirmation. This limitation should be taken into account when interpreting the cell‐type specificity of the S100A8/S100A9 axis in diabetic heart failure. A further limitation of the present study is the absence of human myocardial tissue validation. Although our integrated transcriptomic, single‐cell, and in vitro analyses support an association between the S100A8/S100A9 axis and diabetic heart failure–related inflammatory and fibrotic remodeling, we were unable to confirm these findings directly in human cardiac specimens within the time frame of the current revision. In particular, immunohistochemical or immunofluorescence validation in human myocardial tissue would have substantially strengthened the clinical relevance of the study by verifying both the upregulation of S100A8 and its in situ cellular localization in the diabetic heart. Therefore, the current conclusions regarding clinical translation should be interpreted with appropriate caution. Future studies incorporating human tissue–based spatial and immunophenotypic validation will be important to determine the precise cellular source and pathological distribution of S100A8/S100A9 in diabetic cardiomyopathy.

## Author Contributions

Yang Yang designed the study, carried out the experiments, and drafted the manuscript. Ge Cao conducted data curation, software implementation, and formal analysis.

## Funding

This work was supported by correspondence author.

## Ethics Statement

The authors have nothing to report.

## Consent

The authors have nothing to report.

## Conflicts of Interest

The authors declare no conflicts of interest.

## Supporting information


**Supporting Information** Additional supporting information can be found online in the Supporting Information section. Figure S1:qPCR validation of S100A8 and S100A9 expression following S100A9 knockdown under high‐glucose conditions.

## Data Availability

Data are available from the corresponding author upon reasonable request.

## References

[1] Kunarathnam V. , Vadakekut E. S. , and Mahdy H. , Gestational Diabetes, 2025, StatPearls [Internet] Treasure Island (FL) StatPearls Publishing. 31424780

[2] American Diabetes Association Professional Practice Committee , 7. Diabetes Technology: Standards of Care in Diabetes-2025, Diabetes Care. (2025) 48, no. 1 supplement 1, S146–S166, 10.2337/dc25-S007, 39651978. 39651978 PMC11635043

[3] Cho N. H. , Shaw J. E. , Karuranga S. , Huang Y. , da Rocha Fernandes J. D. , Ohlrogge A. W. , and Malanda B. , IDF Diabetes Atlas: Global Estimates of Diabetes Prevalence for 2017 and Projections for 2045, Diabetes Research and Clinical Practice. (2018) 138, 271–281, 10.1016/j.diabres.2018.02.023. 29496507

[4] Alejandro E. U. , Mamerto T. P. , Chung G. , Villavieja A. , Gaus N. L. , Morgan E. , and Pineda-Cortel M. R. B. , Gestational Diabetes Mellitus: A Harbinger of the Vicious Cycle of Diabetes, International Journal of Molecular Sciences. (2020) 21, no. 14, 10.3390/ijms21145003. PMC7404253 32679915

[5] Crawford A. L. and Laiteerapong N. , Type 2 Diabetes, Annals of Internal Medicine. (2024) 177, no. 6, ITC81–ITC96, 10.7326/AITC202406180. 38857502

[6] Hart P. A. , Bradley D. , Conwell D. L. , Dungan K. , Krishna S. G. , Wyne K. , Bellin M. D. , Yadav D. , Andersen D. K. , Serrano J. , and Papachristou G. I. , Diabetes Following Acute Pancreatitis. Lancet, Lancet Gastroenterology and Hepatology. (2021) 6, no. 8, 668–675, 10.1016/S2468-1253(21)00019-4. 34089654 PMC8277724

[7] Khan R. M. M. , Chua Z. J. Y. , Tan J. C. , Yang Y. , Liao Z. , and Zhao Y. , From Pre-Diabetes to Diabetes: Diagnosis, Treatments and Translational Research, Medicina. (2019) 55, no. 9, 10.3390/medicina55090546. PMC6780236 31470636

[8] Bielka W. , Przezak A. , Molęda P. , Pius-Sadowska E. , and Machaliński B. , Double Diabetes-When Type 1 Diabetes Meets Type 2 Diabetes: Definition, Pathogenesis and Recognition, Cardiovascular Diabetology. (2024) 23, no. 1, 10.1186/s12933-024-02145-x. PMC10859035 38341550

[9] Cole J. B. and Florez J. C. , Genetics of Diabetes Mellitus and Diabetes Complications, Nat Rev Nephrol. (2020) 16, no. 7, 377–390, 10.1038/s41581-020-0278-5. 32398868 PMC9639302

[10] Kenny H. C. and Abel E. D. , Heart Failure in Type 2 Diabetes Mellitus, Circulation Research. (2019) 124, no. 1, 121–141, 10.1161/CIRCRESAHA.118.311371. 30605420 PMC6447311

[11] James S. , Erlinge D. , Storey R. F. , McGuire D. K. , de Belder M. , Eriksson N. , Andersen K. , Austin D. , Arefalk G. , Carrick D. , Hofmann R. , Hoole S. P. , Jones D. A. , Lee K. , Tygesen H. , Johansson P. A. , Langkilde A. M. , Ridderstråle W. , Parvaresh Rizi E. , Deanfield J. , and Oldgren J. , Dapagliflozin in Myocardial Infarction Without Diabetes or Heart Failure, NEJM Evidence. (2024) 3, no. 2, 10.1056/EVIDoa2300286. 38320489

[12] Bhatt D. L. , Szarek M. , Steg P. G. , Cannon C. P. , Leiter L. A. , McGuire D. K. , Lewis J. B. , Riddle M. C. , Voors A. A. , Metra M. , Lund L. H. , Komajda M. , Testani J. M. , Wilcox C. S. , Ponikowski P. , Lopes R. D. , Verma S. , Lapuerta P. , Pitt B. , and Soloist-Whf Trial Investigators , Sotagliflozin in Patients With Diabetes and Recent Worsening Heart Failure, New England Journal of Medicine. (2021) 384, no. 2, 117–128, 10.1056/NEJMoa2030183. 33200892

[13] Park J. J. , Epidemiology, Pathophysiology, Diagnosis and Treatment of Heart Failure in Diabetes, Diabetes and Metabolism Journal.(2021) 45, no. 2, 146–157, 10.4093/dmj.2020.0282. 33813813 PMC8024162

[14] Jankauskas S. S. , Kansakar U. , Varzideh F. , Wilson S. , Mone P. , Lombardi A. , Gambardella J. , and Santulli G. , Heart Failure in Diabetes, Metabolism. (2021) 125, 154910, 10.1016/j.metabol.2021.154910. 34627874 PMC8941799

[15] Wu X. , Liu H. , Brooks A. , Xu S. , Luo J. , Steiner R. , Mickelsen D. M. , Moravec C. S. , Jeffrey A. D. , Small E. M. , and Jin Z. G. , SIRT6 Mitigates Heart Failure With Preserved Ejection Fraction in Diabetes, Circulation Research. (2022) 131, no. 11, 926–943, 10.1161/CIRCRESAHA.121.318988. 36278398 PMC9669223

[16] Lu B. , Wang S. , Wang S. , Cai C. , Wang J. , and Pan L. , Transfer Learning-Based Stiffness Modelling of Spherical Scissor Linkage Remote-Centre-of-Motion Mechanism for Structural Optimisation, International Journal of Medical Robotics and Computer Assisted Surgery. (2026) 22, no. 2, e70143, 10.1002/rcs.70143. 41784104

