# Supplementary figures and images for: S100A8/S100A9 Links Diabetic Stress to Cardiac Progenitor Cell Dysfunction and Fibrotic Heart Failure: An Integrated Transcriptomic, Single‐Cell, and Functional Study
Source: Hum Mutat. 2026 Jul 11;2026:1662522. doi: 10.1155/humu/1662522 (PMC13355290; doi:10.1155/humu/1662522)

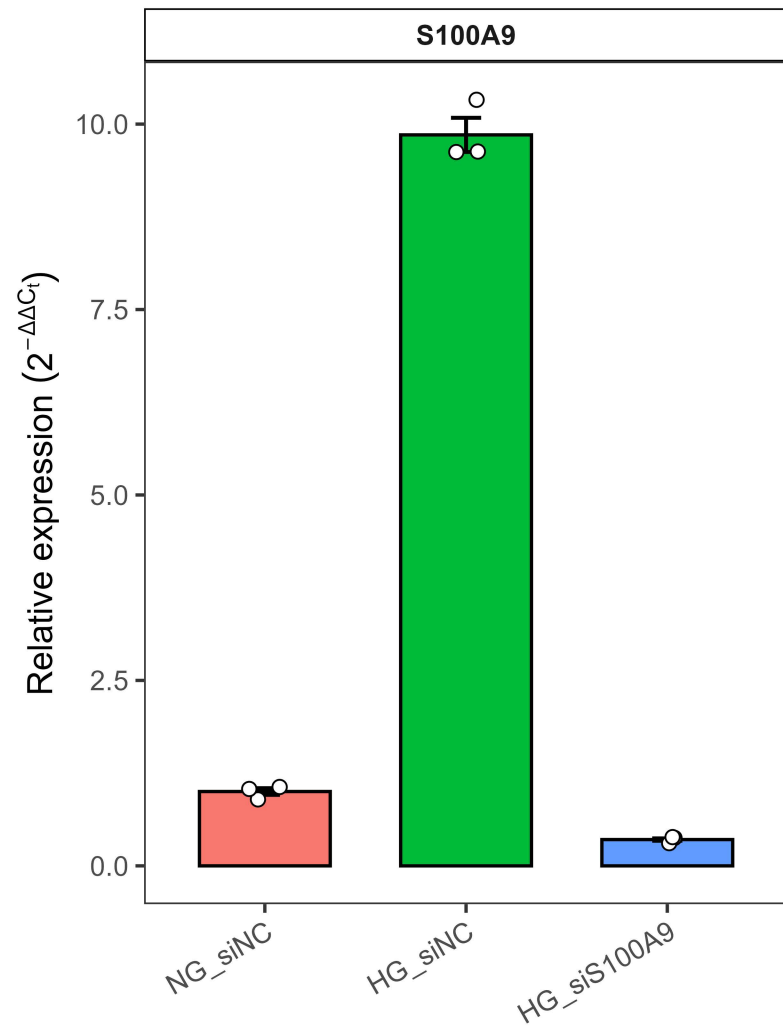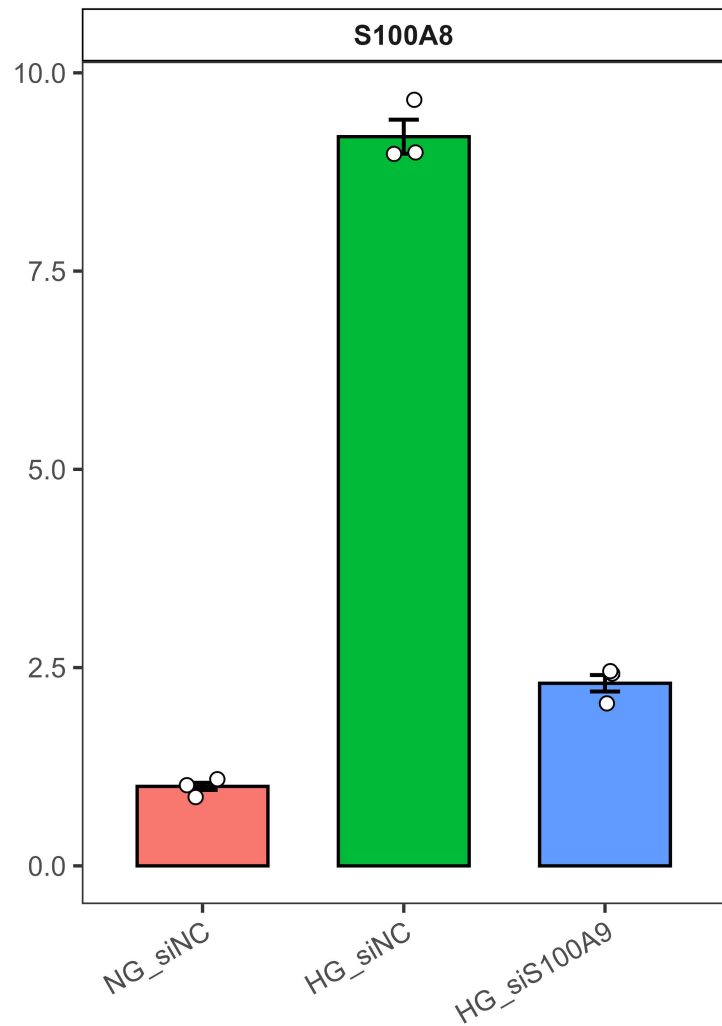

Supplement: Supplementary file 1 — Supporting Information Additional supporting information can be found online in the Supporting Information section. Figure S1:qPCR validation of S100A8 and S100A9 expression following S100A9 knockdown under high‐glucose conditions. [file HUMU-2026-1662522-s001.pdf]
